# Women’s work–family conflict and its consequences in commuter marriages: The moderating role of spouses’ family commitment in a dyad analysis

**DOI:** 10.3389/fpsyg.2022.860717

**Published:** 2022-08-24

**Authors:** Hsin-Pei Wu, Yu-Mei Wang

**Affiliations:** ^1^Department of Business Administration, Soochow University, Taipei City, Taiwan; ^2^Department of Business Administration, Providence University, Taichung, Taiwan

**Keywords:** commuter marriage, family commitment, family satisfaction, job satisfaction, work–family conflicts, marriage satisfaction

## Abstract

This study aims to explore the relationship between work-family conflict and its consequences on job, family, and marital satisfaction among stay-at-home wives of commuter couples by testing the moderating effect of commuters’ family (parental, marital, and household) commitment. The phenomenon of commuter marriages is detectable among well-educated and employed couples in modern society. The study collected dyadic data from 120 dual-earner and noncohabitating couples by using convenience sampling. The analytical approach of the Actor-Partner Interdependence Model was adopted. The results revealed that stay-at-home wives perceived more job dissatisfaction due to work-to-family conflicts and perceived more job, family, and marital dissatisfaction caused by family-to-work conflicts. Moreover, the partner moderating effect of the commuters’ family commitment as spouse support reversed the negative relationship between stay-at-home wives’ family-to-work conflicts and family satisfaction.

## Introduction

Commuter marriages (CMs), also known as dual-career commuter marriages/family/couples (Gross, 1980; Bunker et al., 1992; Jackson et al., 2000; Rhodes, 2002), are marriages between couples living in different residences at least three nights a week (Gerstel and Gross, 1984) or more than 4 days a week (Rabe, 2001) because their workplaces are geographically far from each other. Given the demands of the changing business environment and the fulfillment of career expectations for both men and women, CMs are detectable in many countries such as the United States (McBride and Bergen, 2014), Germany (Reuschke, 2010), Israel (Landesman and Seward, 2013), the Netherlands (van der Klis and Karsten, 2009), Korea (Lee, 2018a), and Hong Kong (Lau et al., 2012). There are ever more CMs in Taiwan.

As Lindemann (2017) posited, the CMs literature on dual-earning professionals who lived apart had been based exclusively on research from the 1970s and 1980s, and many researchers explored the attributions for the expansion and the psychological consequences of these relationships. For example, Gerstel and Gross (1982) found that CMs are an “extension of the individuating tendency in an advanced industrial economy” and that the prevalent ideology of individualism stimulated women’s aspirations for economic and social independence from their husbands. Moreover, Bunker et al. (1992) found that commuters were more satisfied than single-residence couples with their work and their time alone but dissatisfied with their family life and partner relationship. Recently, Lindemann (2017) observed well-educated professionals in CMs and found individualistic tendencies and interdependence among people in CMs. Interestingly, these couples displayed their interdependence by using communication technologies to coordinate family task-sharing and cooperation virtually between partners while also emotionally and logistically maintaining a sense of apart togetherness. This finding implies that commuters could be distant family helpers and facilitate the wellbeing of the stay-at-home spouses, who usually suffer from work–family conflict (WFC) (Green et al., 1999). Research addressing stay-at-home spouses who also have a job (hereafter stay-at-home spouse) in CMs is seemingly scant, especially for improving their wellbeing. Therefore, this research explored the relationship between WFC and work/family/marriage satisfaction among stay-at-home spouses. We also proposed commuters’ family commitment as a dyadic resource from the view of the conservation of resources (COR) theory for moderating these effects.

This research makes some valuable contributions to the CMs field. First, we filled the gap in the literature regarding the lack of quantitative research on CMs mentioned by Murray-Close (2019), especially using non-Western samples (Lee, 2018b; Fallahchai et al., 2019). This study also employed well-educated Taiwanese commuter couples with full-time jobs using two-sided (husband and wife) data. However, this study focused solely on CMs with stay-at-home spouses (wives) and commuters (husbands), which is generally in line with Asian societies and previous studies (c.f. Lee, 2018a). Second, this study was concerned with the overlooked group of stay-at-home spouses (wives) to enrich the current research in the CMs field. Previous research on CMs emphasized the view of commuters including individualism and interdependence (Lindemann, 2017), gender roles (Lindemann, 2018), family division (van der Klis and Karsten, 2009), and the differences in marriage satisfaction between CMs and cohabiting families (Chrishianie et al., 2018; Lee, 2018a). We explored the relationship between WFC and the job/family/marriage satisfaction of the stay-at-home spouses using the actor–partner interdependence model (APIM). Finally, most empirical research over the last three decades has focused on job commitment or employees’ job-related abilities to cope with WFC (c.f. Toscano et al., 2022). However, commuters’ family commitment to supporting their spouses in the family domain should be addressed as indicated above and could be a critical coping resource for stay-at-home spouses in CMs. Therefore, we investigated commuters’ family commitment as a moderator that could buffer the relationship between WFC and the wellbeing of stay-at-home spouses.

## Literature review and hypotheses development

### Conservation of resources theory

The COR theory proposed by Hobfoll (1989) is an integrated theoretical framework that explains how resources are related to stress response (Hobfoll and Shirom, 2001). The COR theory explains how the conflict between different roles leads to stress and the operation mode of resource acquisition and loss in personal and social systems (Hobfoll, 1989). In addition, when individuals suffer a resource loss, such as work or family time reduction, stress will inevitably arise, thus affecting their work and family conditions. Therefore, when an individual is threatened by resource loss or encounters resource loss, they intuitively invest in other resources to prevent or compensate for their loss of valuable resources (Hobfoll and Lilly, 1993).

Moreover, ten Brummelhuis and Bakker (2012) also applied the COR theory to the context of work–family interactions and used resources in their work–home resources model. According to the work–home resources model, work stressors consume employees’ energy, thereby affecting the home domain (Chen and Ellis, 2021). The source of work stress overflows into non-work domains and penetrates non-work hours, making it difficult for an individual to recharge and recycle essential personal resources (Frone et al., 1992). When an individual’s energy is exhausted, they cannot meet their needs and may not be able to build other vital resources.

Both the COR theory and the work–home resources model propose that social support is a unique and important personal resource. “Social support refers to a social network’s provision of psychological and material resources intended to benefit an individual’s ability to cope with stress” (Cohen, 2004, p. 676). Social support, such as emotional support, love, advice, respect, and instrumental support from others, is a vital resource because it helps one solve their situational needs and allows them to preserve valuable resources while preventing further resource loss (Hobfoll, 1989). In addition, the support given by a spouse can help individuals increase their confidence and courage when dealing with stress (Hobfoll, 1989). Moreover, some studies have suggested that social support acts as a buffer against stress or moderates it (e.g., Ganster et al., 1986). Specifically, Berkowitz and Perkins (1984) found that spousal support is vital to the other party and can help alleviate WFC. Falconier et al. (2015) also mentioned that the association between stress and relationship outcomes decreased if the partner provided support. Spousal support in CMs is believed to help partners cope with stress (Lee, 2018a). In other words, the compensation resources, such as the support from one’s spouse, reduce the loss of resources, thereby alleviating WFC.

### Work–family conflict and its consequences for commuter marriage

Work and family are not two independent interfaces but interfere with each other and cause conflicts (Kanter, 1977). Therefore, WFC is often regarded as a kind of stress (Carlson et al., 2000). Couples in CMs share family responsibilities and job demands simultaneously (Krishnan et al., 2020) and require much time and energy to satisfy the work and family domains. As far as CMs are concerned, women tend to bear higher work and life stress levels than men because they need to participate more in family affairs, which consumes significant energy (Puchmüller and Fischlmayr, 2017). According to the COR theory, if individuals experience stress in one domain, they will invest more resources in that domain to reduce stress (Oge et al., 2018). Stay-at-home wives are forced to invest more resources than the husband in the family domain to avoid family failure, but this investment leads to WFC.

Many scholars have suggested distinguishing WFC from work-interface-family conflict (WIF), which occurs when work interferes with family life, and family-interface-work conflict (FIW), which occurs when family life interferes with work life (Carlson et al., 2000). Many studies have investigated ways to reduce WFC (WIF and FIW). For example, Major et al. (2008) explored the impact of work–family culture and leader and coworker relationships on WIF. Montazer and Young (2020) underscored the importance of compensation via positive residential qualities in the relationship between commuting distance and WIF for commuters. Toscano et al. (2022) examined whether FIW mediates the relationship between leader–member exchange and remote working dissatisfaction during the COVID-19 pandemic.

The consequences of WFC can be divided into two categories: work-related and non-work-related (Eby et al., 2005). The former category includes job satisfaction, organizational commitment, turnover intention, absenteeism, job performance, career satisfaction, and career success; the latter contains life satisfaction, wellbeing, family satisfaction, and marriage satisfaction (Chang et al., 2015). The outcomes that have received the most attention in the literature are job satisfaction, family satisfaction, and marriage satisfaction. Job satisfaction is an intrinsic state that evaluates to what degree a person enjoys their work (Brief, 1998), whereas family satisfaction is the quality of home affairs and the results of stress at home (Olson and McCubbin, 1983), and marriage satisfaction is the quality of the marital relationship (Spanier, 1976).

### The relationship between work-interface-family conflict and job/family/marriage satisfaction

Having young children requires especially large time and energy investments, which may exacerbate WIF, especially for mothers who bear most of the parenting responsibilities (Allen and Finkelstein, 2014; Lee, 2018b). For couples with children in CMs, temporary assignments after work may affect the family care tasks of the stay-at-home spouse and the precious leisure time with their family members for commuters, thus leading to job dissatisfaction. Previous studies have shown that WIF and job satisfaction are negatively correlated (Allen et al., 2000, 2020; Hwang and Ramadoss, 2017; Hong et al., 2021).

Moreover, due to the job duties, the stay-at-home spouse cannot meet all family and childcare needs. WIF reduces the lower family quality and family dissatisfaction. In addition, the commuter cannot provide assistance for the stay-at-home spouse, leading to marital dissatisfaction. Exposure to stress in one domain causes tension, fatigue, and irritability, which affect one’s ability to perform in the other domain (Greenhaus and Beutell, 1985). Frone et al. (1992) provided such results by following a cross-domain perspective. According to the work–home resources model, WIF may lead to a feeling of stress and helplessness in one’s family life. As such, Michel et al. (2009) indicated that greater levels of WIF were associated with lower marriage and family satisfaction. In addition, WIF was found to be negatively correlated with marriage satisfaction in Taiwan (Lu et al., 2010). Most studies agree that the burden of raising children deteriorates the working spouse’s role, causing conflict in non-cohabiting couples and increasing marriage dissatisfaction (Challiol and Mignonac, 2005; Sandow, 2014; Lee, 2018b). As such, for stay-at-home women, we predict the following:

Hypothesis 1. WIF is negatively related to job satisfaction (1a), family satisfaction (1b), and marriage satisfaction (1c).

### The relationship between family-interface-work conflict and job/family/marriage satisfaction

Specifically, stay-at-home wives tend to take on the responsibilities of caring for children or relatives, doing household chores, and dealing with discipline problems. These family requirements are possible factors in their work domains (FIW). Brough et al. (2005) opined that there is a stronger relationship between FIW and family satisfaction. In addition, researchers have asserted that FIW is negatively correlated with family satisfaction (Karatepe and Baddar, 2006). For the stay-at-home spouse in a CM, caring for children and doing housework generate stress and require the spouse to expend significant amounts of time and energy in the family domain. Thus, these spouses may need to reduce the time and energy they spend on their jobs, resulting in lower job performance and job dissatisfaction. These spouses, who are unable to get immediate and nearby assistance from commuters, may also experience marriage dissatisfaction. Although a few studies failed to show a significant relationship between FIW and job satisfaction (c.f. Hong et al., 2021), most studies support that FIW is related to job dissatisfaction. For example, Toscano et al. (2022) observed remote working conditions during the COVID-19 pandemic and discovered that FIW is negatively associated with job satisfaction. In sum, for stay-at-home women, we predict the following:

Hypothesis 2. FIW is negatively related to job satisfaction (2a), family satisfaction (2b), and marriage satisfaction (2c).

### The partner’s family commitment as a buffer

Commitment is one’s willingness to expend their personal temporal and psychological resources on behalf of a particular domain that satisfies their needs and values (Mowday et al., 1982; Amatea et al., 1986). This study focused on commitment to three commonly held roles in the family: the marital role, the parental role, and the homecare role. When one’s spouse is willing to invest considerable time and energy in the family domain, they have a high family commitment. Sawai et al. (2020) showed that commitment is positively related to marriage maintenance among teachers in CMs. Hence, for this study, family commitment was considered a vital factor.

In marriage and family life, living with a partner is regarded as a conditional resource for preventing strain (Vachon, 1986). However, stay-at-home spouses may be overworked at work while lacking the commuter’s help with chores and childcare responsibilities, or the partner does not immediately share the stay-at-home spouse’s emotional exhaustion. The stay-at-home spouse lacks the physical and psychological support required to maintain their lives and cope with the strain. In the case of resource loss, resource acquisition is important (Hobfoll and Lilly, 1993). According to the COR theory, acquiring more resources, such as the support of a spouse, whether substantive or emotional assistance, reduces the loss of resources, thereby alleviating the WFC. When the stay-at-home spouse feels the resource loss, personal resource acquisition is critical as a kind of social support. That is, the acquisition of personal resources can buffer the relationship between WFC and its consequences. We suggest that family commitment is a resource that can become a potential investment and social support for the stay-at-home spouse to buffer the strain of temporarily losing the other party.

For a stay-at-home spouse, the actor effect is the moderating effect of their family commitment on the relationship between WFC and job/family/marriage satisfaction. The partner effect refers to the moderating effect of their partner’s family commitment on the relationship between WFC and job/family/marriage satisfaction. When the stay-at-home spouse finds that the commuter has strong family commitments, they assume that the commuter’s concern with their family and marriage is a form of mental support. This may weaken the relationship between WFC and job/family/marriage satisfaction. As such, for stay-at-home women, we propose the following:

Hypothesis 3. The partner’s family commitment weakens WIF’s relationships with job satisfaction (H3a), family satisfaction (H3b), and marriage satisfaction (H3c).

Hypothesis 4. The partner’s family commitment weakens FIW’s relationships with job satisfaction (H4a), family satisfaction (H4b), and marriage satisfaction (H4c).

**FIGURE 1:**
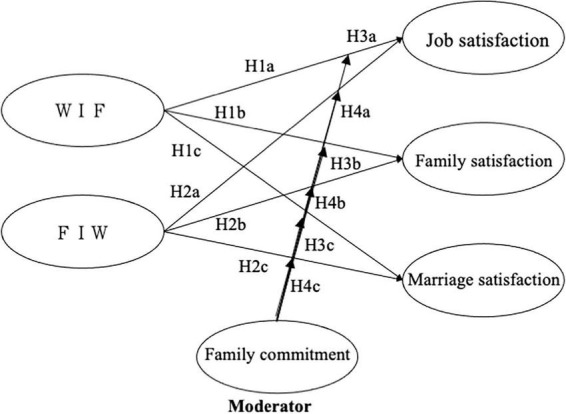
The conceptual model.

Figure 1 depicts our conceptual research model and hypotheses.

## Materials and methods

### Participants and procedures

Participants were eligible for the study if they were in non-cohabiting, heterosexual, and dual-earner married couples raising at least one child. As discussed, commuter couples have to live apart from their spouses because of the demands of their jobs at least 4 days a week. Also, each partner had to be employed full time and take responsibility for raising the children to highlight the strains of both the work and family domains.

Due to the difficulty of practicing random sampling, we adopted a purposive sampling method for recruiting qualified participants through personal contacts. Nevertheless, we aimed to vary our sample by considering the heterogeneity of different industries, organizations, job positions, and ages. While potential couples fit these selection criteria, we asked personal contacts to deliver a package with a cover letter, two hard copy questionnaires with different colors to identify husband and wife versions, and two envelopes. The questionnaire content for couples was identical, and items were generated for people to self-report staying/leaving home and their family and work locations (counties and cities) for final confirmation. The couples were asked to complete the questionnaires independently without discussion. The stay-at-home spouse would keep the commuter’s questionnaire for them to complete upon returning home. Each spouse had a separate envelope for confidentiality, which they returned to the contact person or mailed to the researchers. They could also scan a digital copy and forward it to the researchers separately due to the previous stamping of the representative numbers for each couple.

In total, 328 samples were received over almost 1 year in 2017. We checked each couple on the family location, which is also the wife’s work location but different from the husband’s. After removing couples who had no children, female commuters, unidentified locations, or who returned only one questionnaire, 120 (240 respondents) couples were included. The valid return rate was 73.17%.

The characteristics of stay-at-home female spouses included age (*M* = 40.17, SD = 7.40), years of formal education (*M* = 15.15, SD = 2.33, 74% a bachelor’s degree or above), organization tenure (*M* = 10.30, SD = 8.56), job position (84.2% non-managers), daily working hours (*M* = 8.65, SD = 2.37), and extended working hours per day (*M* = 1.77, SD = 3.08). The male commuters had an average age of 41.97 years (SD = 9.30), an average of 15.9 academic years (SD = 1.97, 87.5% a bachelor’s degree or above), and an average organization tenure of 13.51 years (SD = 8.94). Furthermore, 65.5% of them held a managerial position, their daily working hours was 10.11 h (SD = 2.47), and their extended working hours per day were 1.61 h (SD = 2.64).

The family background comprised the number of children (*M* = 1.86, SD = 0.6), children’s age (*M* = 9.95, SD = 7.59), average marriageable age (*M* = 12.32, SD = 7.98), and years of non-cohabitation (*M* = 5.84, SD = 5.58). It took an average of 23 days for each couple to meet each other (SD = 0.87). In total, 71.3% of couples did not live with their parents (or parents-in-law), and 8.4% of them indicated that they lived near their parents. Finally, 26 (21.67%) commuters were expatriates.

### Measures

#### Work–family conflict

We adopted a 10-item WFC scale from Netemeyer et al. (1996) to measure personal experiences on the interference of paid work and family life. Five items are related to WIF, such as “The demands of my work interfere with my home and family life,” and five items address FIW, such as “The demands of my family or spouse/partner interfere with work-related activities.” Responses ranged from 1 (*strongly disagree*) to 7 (*strongly agree*). We averaged the items to create an index, with higher scores indicating more WFC (WIF and FIW).

#### Job satisfaction

We used a three-item job satisfaction scale based on the Michigan Organizational Assessment Questionnaire (Cammann et al., 1979). One example item is “Overall, I am satisfied with my work.” Responses ranged from 1 (*strongly disagree*) to 6 (*strongly agree*). We coded and averaged items so that higher scores represented greater job satisfaction.

#### Family satisfaction

We employed a three-item scale from Edwards and Rothbard (1999). One example item is “Overall, I am satisfied with my family.” Responses range from 1 (*strongly disagree*) to 6 (*strongly agree*). We coded and averaged the items, with higher scores reflecting higher family satisfaction.

#### Marriage satisfaction

We measured marriage satisfaction using Spanier’s (1976) dyadic satisfaction subscales. Four items were included: “How often do you discuss or have you considered divorce, separation, or terminating your relationship?” “In general, how often do you think that things between you and your partner are going well?” “Do you confide in your mate?” “Do you ever regret that you married?” Responses ranged from 0 (*all the time*) to 5 (*never*). A higher average score represented greater marriage satisfaction.

#### Family commitment

We adopted marital, parental, and homecare roles commitment to measure family commitment using the life role salience scale by Amatea et al. (1986). Eight items were chosen by factor loadings for use. The two items for measuring parental role commitment were “I expect to devote a significant amount of my time and energy to the rearing of children of my own” and “Becoming involved in the day-to-day details of rearing children involves costs in other areas of my life which I am unwilling to make (reversed item).” Three items were used to assess marital role commitment: “I expect to commit whatever time is necessary to making my marriage partner feel loved, supported, and cared for,” “Devoting a significant amount of my time to be with or doing things with a marriage partner is not something I expect to do (reversed item),” and “I expect to work hard to build a good marriage relationship with opportunities to pursue other personal goals.” Another three items were related to homecare role commitment: “I expect to be very much involved in caring for a home and making it attractive,” “I expect to be very much involved in caring for a home and making it attractive,” and “I expect to leave most of the day-to-day details of running a home to someone else (reversed item).” Responses for all items ranged from 1 (*strongly disagree*) to 5 (*strongly agree*). Higher average scores reflected stronger family commitment.

### Analysis strategy

The actor-partner interdependence model (Kashy and Kenny, 2000) allows researchers to simultaneously examine the actor effect of one’s own predictor score on one’s own outcome and the partner effect on the outcome of one’s partner. We adopted structural equation modeling (SEM) procedures to fit the proposed model. The SEM framework has advantages over multilevel models (MLM). First, it allows constraining the mean and variance of the independent variables and covariates to be equal across roles. Second, it relatively and easily allows for the unreliability of predictors. Third, the full information maximum likelihood (FIML) by default is used to estimate the missing data (Stas et al., 2018).

Two user-friendly online apps–APIM_SEM (Stas et al., 2018; freely accessible at)^1^ and APIMoM (Kenny, 2015a; available from) – were used to automatically perform the statistical analyses based on the program lavaan (Rosseel, 2012).^2^ Following the APIM apps procedures, we adopted a basic APIM enrolling only one independent variable (but from both roles counting the actor and partner effects at the same time) into the model and labeled the stay-at-home spouses (wives) as Role 1 and the commuters (husbands) as Role 2 to achieve an identifiable model. All predictors were grand mean centered by the apps to reduce the potential issue of multicollinearity. While we tested the moderating effects, standardized estimates used pooled variances across members, and the standardization for interaction effects used the product of the two standard deviations. All figures were automatically generated from the APIMoM program but revised slightly for clarity.

Before testing the hypotheses, we evaluated the model fit for confirmatory factor analysis (CFA) using the following indices: chi-square, comparative fit index (CFI), Tucker–Lewis index (TLI), and root mean square error of approximation (RMSEA). We considered CFI and TLI values of 0.95 or above indicative of an excellent fit and RMSEA values of 0.08 or below indicative of a good fit (Hu and Bentler, 1998). Moreover, tests of distinguishability and non-independence were examined in Dingy, a computer software program available from^3^ (Kenny, 2015b). The chi-square test, the chi-square difference test, RMSEA, and the sample size-adjusted Bayesian information criterion (SABIC) were adopted to compare models. The SABIC is a badness-of-fit index, for which smaller values indicate a better fit. One advantage of the SABIC is that a value can be computed for full distinguishability even though it is a saturated model with zero degrees of freedom.

## Results

### Reliability, validity, and correlations

Table 1 presents the means, standard deviations, Cronbach’s alpha coefficients, and correlations of all variables. First, the results of paired *t*-test for comparing differences in our main variables between commuters and stay-at-home spouses revealed that the commuters perceived more WIF and marriage satisfaction than the stay-at-home spouses. Second, Cronbach’s alpha coefficients for internal consistency reliability were good (α = 0.75) to excellent (α = 0.95) for each variable. Third, CFA was conducted to assess the convergent and discriminant validity of the measures. The model fit indexes of the CFA showed that the six-factor measurement models for both the stay-at-home spouses and commuters samples fit well with the data [stay-at-home spouses: χ^2^ (261) = 381.97, *p* = 0.000, CFI = 0.94, TLI = 0.93, RMSEA = 0.06 and commuters: χ^2^ (261) = 402.32, *p* = 0.000, CFI = 0.92, TLI = 0.91, RMSEA = 0.07]. Regarding convergent validity, the factor loadings of the indicators for each construct were almost greater than 0.5 and statistically significant for both samples (stay-at-home spouses: from 0.46 to 0.99; commuters: from 0.47 to 0.96; Hair et al., 2006).^4,5^ Because the FIW variable (for commuters) and the family commitment variable (for stay-at-home spouses) were not the independent variables involved in testing our hypotheses, factor loadings (standardized regression weights) slightly less than 0.5 could be acceptable. Also, the corrected correlations of each latent variable for stay-at-home spouses from 0.02 to 0.79, less than ± 0.85, as suggested by Kenny (2011), indicated good discriminant validity but not for the sample of commuters.^6,7^ Although discriminant validity was weak between the dependent variables for commuters, we tested our model with these dyadic data since the hypotheses mainly involved the dependent variables of the stay-at-home spouse sample. Finally, most of the relationships between the studied variables were significant and took the expected directions. For controls, we added potential covariates that were significantly related to our independent variables. Specifically, we added the stay-at-home spouses’ position and meet period as controls for predicting family satisfaction and the commuters’ age, marriageable age, and children’s age for predicting job satisfaction.

**TABLE 1:** Mean, standard deviation, Cronbach’s alpha coefficients, and inter-correlations for variables.

Variables	S^a^: Mean (SD)	S: α	1	2	3	4	5	6	7	8	9	10	11	12	13	14	15	16
C^b^: Mean (SD)	–	–	41.97 (9.30)	15.9 (1.97)	0.66 (0.48)	13.51 (8.94)	12.32 (7.98)	1.86 (0.6)	9.95 (7.59)	0.29 (0.45)	5.84 (5.58)	0.77 (0.87)	4.56 (1.49)	2.99 (1.12)	4.56 (1.07)	5.13 (0.86)	4.22 (0.66)	4.06 (0.61)
C: α	–	–	–	–	–	–	–	–	–	–	–	–	0.93	0.82	0.75	0.94	0.75	0.80
1. Age	40.17 (7.4)	–	1	−0.26**	0.23*	0.66***	0.80***	0.43***	0.78***	–0.15	0.50***	0.04	–0.02	–0.11	0.23*	0.08	–0.06	0.11
2. Education	15.15 (2.33)	–	–0.17	1	–0.04	−0.21*	−0.35***	–0.06	−0.37***	0.03	−0.49***	−0.29**	–0.05	0.08	0.06	0.10	0.17	0.13
3. Position	0.16 (0.37)	–	0.19*	0.13	1	0.27**	0.28**	0.18	0.27**	−0.21*	0.26**	0.24**	0.14	–0.10	0.06	–0.15	–0.10	0.10
4. Tenure	10.30 (8.56)	–	0.50***	0.01	0.25*	1	0.73***	0.42***	0.72***	–0.17	0.39**	0.06	0.07	0.00	0.14	0.00	–0.08	0.04
5. Marriageable age	12.32 (7.98)	–	0.88***	−0.29**	0.22*	0.47***	1	0.59***	0.98***	–0.15	0.59***	0.15	0.02	–0.18	0.19*	0.05	–0.12	0.11
6. Children	1.86 (0.6)	–	0.42***	−0.22*	0.17	0.25**	0.59***	1	0.55***	0.05	0.23	–0.05	0.07	–0.05	0.16	0.08	–0.04	0.14
7. Children’s age	9.95 (7.59)	–	0.88***	−0.33***	0.16	0.56***	0.98***	0.55***	1	–0.13	0.59***	0.15	0.04	–0.15	0.21*	0.04	–0.16	0.08
8. Living with parents	0.29 (0.45)	–	−0.21*	–0.02	–0.06	–0.18	–0.15	0.05	–0.13	1	–0.21	–0.06	–0.09	–0.18	0.13	–0.01	0.07	0.02
9. Non-cohabitation	5.84 (5.58)	–	0.55***	–0.22	–0.09	0.14	0.59***	0.23	0.59***	–0.21	1	–0.17	0.08	–0.01	0.17	–0.10	–0.09	0.15
10. Meet period	0.77 (0.87)	–	0.09	−0.30***	0.01	–0.06	0.15	–0.05	0.15	–0.06	–0.17	1	0.15	−0.20*	–0.15	–0.09	–0.06	–0.03
11. WIF	3.82 (1.34)	0.93	−0.26**	0.10	–0.07	–0.03	−0.22*	–0.11	−0.26**	–0.01	–0.18	–0.01	*t* = 4.31***	0.26**	−0.33***	–0.07	–0.01	0.03
12. FIW	3.01 (1.09)	0.82	−0.25**	−0.20*	–0.10	–0.10	–0.16	–0.02	–0.17	–0.04	–0.13	0.04	0.55***	*t* = −0.10	–0.02	–0.11	−0.18*	−0.24**
13. Job Satisfaction	4.69 (1.05)	0.80	0.17	–0.05	0.02	0.13	0.12	0.16	0.10	0.06	–0.06	0.04	−0.25**	−0.28**	*t* = −1.00	0.17	0.00	0.10
14. Family Satisfaction	4.98 (1.05)	0.95	0.11	0.04	0.20*	0.15	0.03	0.01	–0.01	–0.12	0.10	−0.23*	–0.06	−0.23*	0.19*	*t* = 1.72	0.64***	0.40***
15. Marriage Satisfaction	4.09 (0.81)	0.80	0.07	0.12	0.08	0.02	0.01	–0.01	–0.04	–0.08	0.10	–0.03	–0.02	−0.20*	0.05	0.55***	*t* = 2.67**	0.41***
16. Family Commitment	4.14 (0.57)	0.78	0.20*	0.04	0.04	0.18	0.19*	0.27**	0.14	–0.03	0.00	–0.03	0.03	−0.21*	0.08	0.37***	0.39***	*t* = −0.93

*n* = 240, 120 couples. ^a^: S stands for stay-at-home spouses; ^b^: C stands for commuters; Bivariate correlations among commuters are shown above the diagonal. Bivariate correlations among stay-at-home spouses are shown below the diagonal. The paired *t*-test values are in the diagonal. α = Cronbach’s alpha coefficients. Education: years of formal education; Position: 0 = non-managers, 1 = managers; Tenure: years of current organizational tenure; Children: numbers of children; Living with parents: 0 = no, 1 = yes; Non-cohabitation: years of non-cohabitation; Meet period: monthly, *p < 0.05, **p < 0.01, ***p < 0.001.

### Testing distinguishability and non-independence

Distinguishability is vital to any dyadic data analysis because empirical tests of distinguishability do not always concord with the natural distinction (e.g., gender; Fitzpatrick et al., 2016). We conducted a distinguishability test across the means, variances, and correlations on the six pairs of variables. The six pairs of key variables had unequal means [χ^2^ (6) = 30.15, *p* < 0.001], unequal variances [χ^2^ (6) = 16.09, *p* = 0.013], and equal correlations [χ^2^ (30) = 40.27, *p* = 0.100]. Thus, we cannot conclude that our data are distinguishable by gender. As a result, we treated dyad members as indistinguishable.

Additionally, we tested 36 correlations between the variables from stay-at-home spouses and commuters. With the dyad members treated as indistinguishable, the non-independence test showed that all six variables were correlated with a better fit [χ^2^ (21) = 150.05, *p* < 0.001; RMSEA = 0.226; SABIC = 193.95; SABIC (Sat) = 78.05] compared with distinguishable, establishing that our dyad data are indeed interdependent and require the APIM.

### Hypotheses testing

Tables 2, 3 present the APIM results for stay-at-home spouses. We displayed the different actor and partner effects of WIF on the dependent variable of only the stay-at-home spouses in each model. However, each model included all actor and partner effects from both roles. The actor effect for Hypothesis 1a was supported, while the actor effects for Hypotheses 1b and 1c were not supported. For Hypothesis 1a, the actor effect of WIF on the job satisfaction of stay-at-home spouses was −0.18 and significant, *p* = 0.01, 95% CI (−0.32, −0.04), with a standardized effect of −0.23. For Hypotheses 1b and 1c, the actor effect of WIF on the family satisfaction of stay-at-home spouses was −0.04 and non-significant, *p* = 0.57, 95% CI (−0.18, 0.10) and the actor effect of WIF on the marriage satisfaction of stay-at-home spouses was −0.05 and non-significant, *p* = 0.80, 95% CI (−0.12, 0.10), respectively.

**TABLE 2:** The APIM results from WIF to dependent variables of stay-at-home spouses.

Variables	Model 1: Job satisfaction				Model 2: Family satisfaction				Model 3: Marriage satisfaction			
	Estimate	Beta (s)	95% CI		Estimate	Beta (s)	95% CI		Estimate	Beta (s)	95% CI	
			Lower	Upper			Lower	Upper			Lower	Upper
WIF: actor	−0.18**	−0.23	−0.32	−0.04	−0.04	−0.05	−0.18	0.10	−0.01	−0.02	−0.12	0.10
WIF: partner	−0.05	−0.07	−0.17	0.08	0.07	0.10	−0.06	0.20	0.03	0.06	−0.07	0.13

We only displayed the different actor and partner effects of WIF on the dependent variable of stay-at-home spouses in each model; however, each model included all actor and partner effects from both roles. Beta (s) uses the standard deviation for two samples separately. Three between-dyad covariates (age, marriageable age, and children’s age) were included in Model 1 and non-significant for predicting job satisfaction of both roles. Two between-dyad covariates (position and meet period) were included in Model 2. The effects of the position and meet period on family satisfaction of stay-at-home spouses were statistically significant [position: 0.36, *p* = 0.03, 95% CI (0.03, 0.68); meet period; −0.27, *p* = 0.01, 95% CI (−0.49, −0.01)], and the both effects were non-significant for predicting commuters’ family satisfaction, **p < 0.01.

**TABLE 3:** The APIM results from FIW to dependent variables of stay-at-home spouses.

Variables	Model 4: Job satisfaction				Model 5: Family satisfaction				Model 6: Marriage satisfaction			
	Estimate	Beta (s)	95% CI		Estimate	Beta (s)	95% CI		Estimate	Beta (s)	95% CI	
			Lower	Upper			Lower	Upper			Lower	Upper
FIW: actor	−0.26**	−0.27	−0.43	−0.09	−0.20*	−0.20	−0.36	−0.02	−0.14*	−0.18	−0.27	−0.01
FIW: partner	0.02	0.02	−0.15	0.18	−0.02	−0.02	−0.20	0.16	−0.05	−0.07	−0.18	0.08

We only displayed the different actor and partner effects of WIF on the dependent variable of stay-at-home spouses in each model; however, each model included all actor and partner effects from both roles. Beta (s) uses the standard deviation for two samples separately. Three between-dyad covariates (age, marriageable age, and children’s age) were included in Model 4 and non-significant for predicting job satisfaction of both roles. Two between-dyad covariates (position and meet period) were included in Model 5. The effect of the meet period on family satisfaction of stay-at-home spouses was −0.23, *p* = 0.04, 95% CI (−0.45, −0.01), and the remaining effects were non-significant for predicting both roles’ family satisfaction, *p < 0.05, **p < 0.01.

The partner effects for Hypotheses 2a, 2b, and 2c were all supported. For Hypothesis 2a, the actor effect of FIW on the job satisfaction of stay-at-home spouses was −0.26 and significant, *p* = 0.002, 95% CI (−0.43, −0.09), with a standardized effect of −0.27. For Hypothesis 2b, the actor effect of FIW on the family satisfaction of stay-at-home spouses was −0.20 and significant, *p* = 0.03, 95% CI (−0.36, −0.02), with a standardized effect of −0.20. For Hypothesis 2c, the actor effect of FIW on the marriage satisfaction of stay-at-home spouses was −0.14 and significant, *p* = 0.039, 95% CI (−0.27, −0.01), with a standardized effect of −0.18.

Because negative relationships between WIF and job satisfaction and between FIW and job/family/marriage satisfaction were supported for stay-at-home spouses, we examined the moderating effects of commuters’ family commitment only on these relationships. Before testing Hypotheses 3, 4a, 4b, and 4c, we examined whether the dyad members are distinguishable or not. The chi-square tests for the constraints of equal FIW, family commitment, and their interactions in all testing models were not statistically significant and had RMSEA values of 0.00–0.05, which are less than 0.10. Thus, treating the effects as equal across members is appropriate.

Hypothesis 3 proposed that the relationship between WIF and the job satisfaction of stay-at-home spouses is weaker when the commuters’ family commitment is higher. We tested for moderations in this model. However, the comparison between one model with interaction effects and one model without such effects did not reveal any significant moderations [χ^2^ (4) = 4.66, *p* = 0.32, RMSEA = 0.04].

Hypotheses 4a, 4b, and 4c predicted that the actor effect of FIW on the job/family/marriage satisfaction of stay-at-home spouses is weaker when commuters’ family commitment is higher. However, when we tested Hypothesis 4a, the result of the comparison test indicated that there was no evidence of moderations, χ^2^ (4) = 3.78, *p* = 0.44, RMSEA = 0.00. Moreover, there was no evidence to support moderations for Hypothesis 4c, χ^2^ (4) = 4.42, *p* = 0.35, RMSEA = 0.03. The comparison test was supported only for Hypothesis 4b, χ^2^ (4) = 20.22, *p* < 0.00, RMSEA = 0.18.

Therefore, we present the results of the moderating effects for Hypothesis 4b in Table 4. The table provides the results of the moderating model involving both the actor and partner effects of FIW and family commitment and their combined interactions in affecting family satisfaction in both roles. Hypothesis 4b predicted that the commuters’ family commitment could buffer the relationship between FIW and family satisfaction for stay-at-home spouses. Therefore, the actor–partner interaction term of the actor effect of FIW for stay-at-home spouses and the partner effect of family commitment for commuters should be addressed. The results revealed that the actor–partner interaction term was significantly related to the family satisfaction of stay-at-home spouses, β = 0.18, *p* = 0.003, 95% CI (0.09, 0.43).

**TABLE 4:** The moderating model for predicting family satisfaction.

Variables	Type	Estimate	95% CI		*P*-value	Standardized estimate
			Lower	Upper		
FIW	Actor	−0.06	−0.15	0.04	0.223	−0.04
	Partner	−0.01	0.14	0.09	0.917	−0.00
Family commitment	Actor	0.47	0.30	0.64	<0.001	0.54
	Partner	0.31	0.14	0.48	<0.001	0.35
Interaction	Actor–Actor	−0.33	−0.49	−0.17	<0.001	−0.23
	Actor–Partner	0.26	0.09	0.43	0.003	0.18
	Partner–Actor	0.29	0.12	0.47	<0.001	0.21
	Partner–Partner	−0.14	−0.31	0.03	0.099	−0.10

**FIGURE 2:**
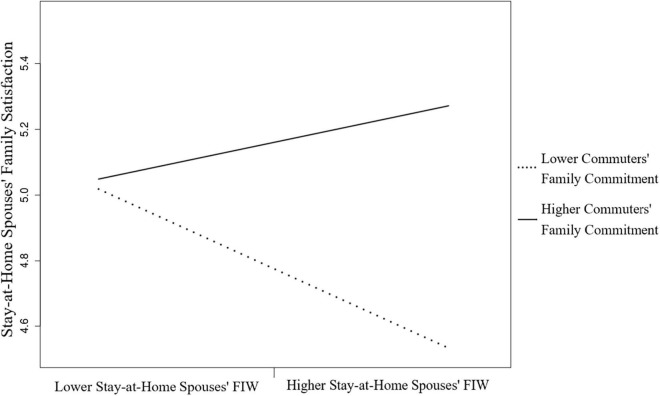
The moderating effect of commuter’s family commitment of the FIW and family satisfaction relation for stay-at-home spouses.

Figure 2 demonstrates the positive relationship between FIW and family satisfaction for stay-at-home spouses when commuters’ family commitment is high (+ 1 SD). The commuters’ family commitment reversed the family dissatisfaction felt by stay-at-home spouses due to FIW. Thus, Hypothesis 4b was supported.

### Supplementary material

We briefly disclosed the APIM results of the commuters and delineated the other two types of significant moderating effects from the results in Table 4. Specifically, WIF was significantly negatively related to job satisfaction for commuters, β_(s)_ = −0.33, *p* < 0.001, 95% CI (−0.35, −0.11). All other actors and partner effects for predicting the three dependent variables of the commuters were non-significant.

**FIGURE 3:**
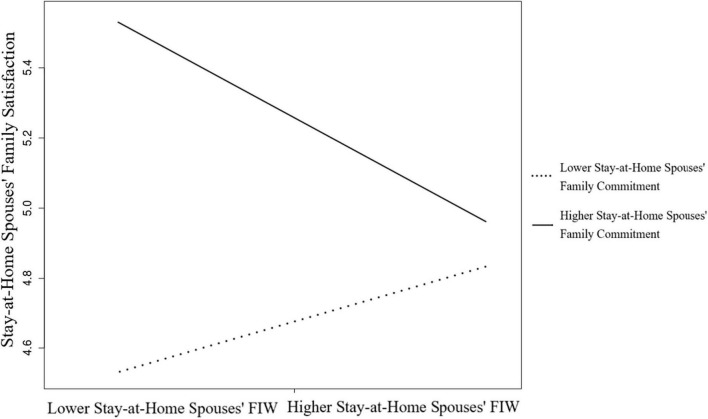
The moderating effect of stay-at-home spouses’ family commitment of the FIW and family satisfaction relation.

Furthermore, the actor–actor moderating effect between actor FIW and actor family commitment on family satisfaction for stay-at-home spouses was significant, β = −0.23, *p* < 0.001, 95% CI (−0.49, −0.17). This result indicates that the family commitment of stay-at-home spouses moderated their FIW–family satisfaction relationship. As Figure 3 illustrates, the slope of the lower (−1 SD) family commitment was positive, which means that when stay-at-home spouses committed to the family less, they experienced higher family satisfaction from FIW.

**FIGURE 4:**
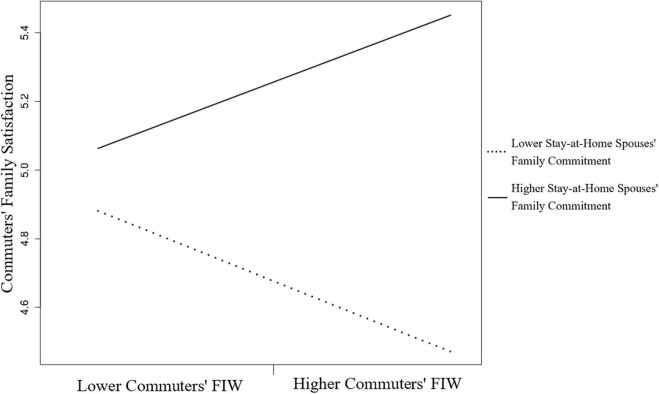
The moderating effect of stay-at-home spouses’ family commitment of the FIW and family satisfaction relation for commuters.

Moreover, the partner–actor interaction term of the partner effect of FIW for commuters and the actor effect of family commitment for stay-at-home spouses on commuters’ family satisfaction was significant, β = 0.21, *p* < 0.001, 95% CI (0.12, 0.47). Figure 4 shows simple slopes at different moderator values of stay-at-home spouses depicting the FIW–family satisfaction relationships of commuters. The slope for the higher (+ 1 SD) stay-at-home spouses’ family commitment was positive. The result indicates that when stay-at-home spouses committed to the family more, the commuters experienced more family satisfaction from their FIW.

**FIGURE 5:**
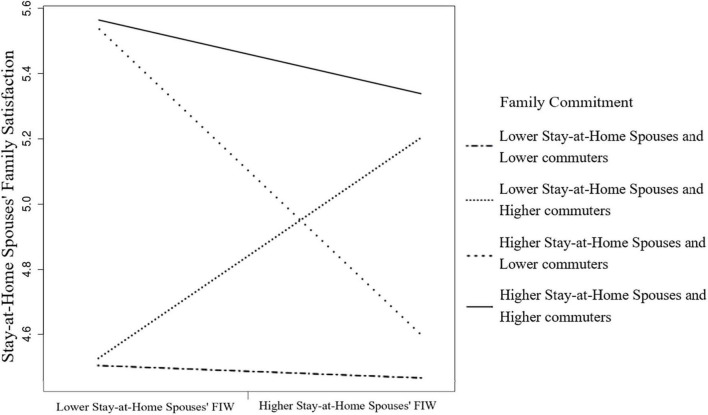
Four combinations of family commitment on stay-at-home spouses’ FIW and family satisfaction relation.

**FIGURE 6:**
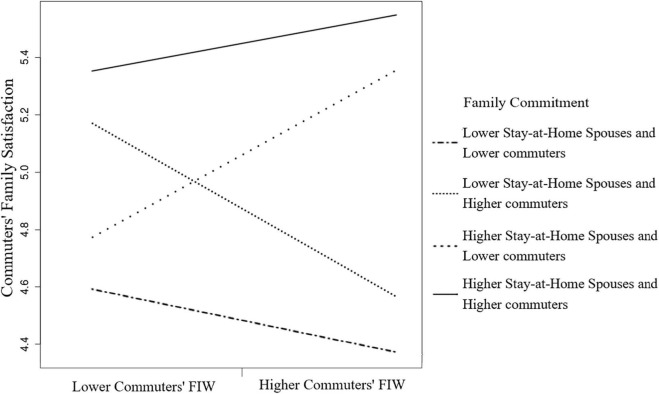
Four combinations of family commitment on commuters’ FIW and family satisfaction relation.

We then illustrated the four combinations of moderating effects on the FIW–family satisfaction relationship. The family commitment combination of the lower stay-at-home spouses (−1 SD) and the higher commuters (+1 SD) situation had a positive slope on the FIW–family satisfaction association of stay-at-home spouses (Figure 5). The combination of the higher stay-at-home spouses (+ 1 SD) and the lower commuters (−1 SD) situation had the steepest and positive slope on the FIW–family satisfaction relation of commuters in Figure 6. The results shown in Figures 5, 6 indicate that individuals involved in FIW had more family satisfaction when their spouses committed to more family roles than they did.

Overall, both the stay-at-home spouses committed to fewer family issues and their partners committed more upturned stay-at-home spouses’ family dissatisfaction due to FIW. However, when stay-at-home spouses commit to family more, the commuters feel more family satisfaction even though the significant main effect of FIW–family satisfaction association for commuters was not found in our study.

## Discussion

Our study answers the call to provide empirical evidence regarding promoting the work–family balance in dual-career commuting families (c.f. Känsälä et al., 2015; Mutter and Thorn, 2018). This study explored the relationships between WFC and the wellbeing of stay-at-home spouses (wives), who suffer more role conflicts than commuters (husbands), as indicated by previous studies (van der Klis and Karsten, 2009; Lindemann, 2018) in dual-career commuting families. We employed the idea of resource compensation from the COR theory and proposed commuters’ family commitment as a partner moderator that buffers the intense harm caused to wives’ wellbeing by WFC.

We collected dyad data from both roles, and the results confirmed that stay-at-home spouses who experienced more WFC perceived lower satisfaction. Specifically, stay-at-home wives perceived job dissatisfaction caused by WIF and perceived job, family, and marriage dissatisfaction due to FIW. Stay-at-home spouses might perceive non-cohabitation as a family demand that undermines their family life and even their work, even though our female sample had maintained the non-cohabitation lifestyle for around five years on average.

Even though WFC has been examined in a number of studies as a factor influencing job, family, and overall life satisfaction (Ernst Kossek and Ozeki, 1998; Allen et al., 2000; Ford et al., 2007), the negative associations between WIF and family satisfaction and between WIF and marriage satisfaction were not supported in our stay-at-home wives. Känsälä et al. (2015) found that the partners of frequent international travelers utilize part-time employment to manage the inter-role conflict they experience between their work and home domains. Similarly, we found that stay-at-home wives had fewer weekly working hours on average than commuters, although wives were also employed full time. The wives in our study also experienced less WIF than their husbands. Stay-at-home wives seemingly tried their best to avoid WIF, or they might have adopted resources to deal with WIF’s influence on the vulnerable family domain and marital relationship in advance.

Moreover, couples were likely to coordinate their career development and family responsibility. As dual-career expatriate couples, they employed the hierarchical strategy (the leading career is prioritized over the secondary career and the couple’s relationship) to arrange their lives (Känsälä et al., 2015). Therefore, WIF had no destructive effects on the stay-at-home wives’ wellbeing in the family domain in our study.

Additionally, commuters (husbands) with higher WIF experienced more job dissatisfaction. Commuters might relate non-cohabitation to work demands, which, in turn, leads to negative feelings about their jobs. However, no other significant associations were discovered. Consequently, family roles (commuter/stay-at-home) in different family conditions (cohabitation/non-cohabitation) partly explain WFC and its consequences.

More importantly, we found that when commuters were more committed to their families, stay-at-home wives displayed more family satisfaction despite high FIW. The resources gained from husbands’ support reduced stay-at-home wives’ strain when interference depleted the wives’ resources. This finding reflects the concept of conjugal interdependence in Lindemann’s (2017) qualitative study, although we do not know how the commuters in our sample committed to their family tasks. However, the commuters might engage in parenting responsibilities and carry out straightforward household tasks during weekends or days when they are at home, as suggested by a study in the Netherlands (van der Klis and Karsten, 2009). Commuters could also share family tasks online, as Lindemann (2017) indicated.

Moreover, the stay-at-home wives who committed less family commitment buffered their FIW–family satisfaction relationship. The husbands in our sample perceived more family satisfaction due to FIW when stay-at-home wives were involved in more family issues. Whether the family commitment combination of the higher wives and the lower husbands for predicting husbands’ family satisfaction or the combination of the lower wives and the higher husbands for wives’ family satisfaction indicated the most buffered effect, we confirmed the compensatory effect of resources by disentangling the joint effects of spouse resources. However, the high family commitment situations (see Figures 4, 5) showed the highest level of family satisfaction for both roles. It seems that family commitment driven by conjugal consensus might be beneficial for all members of the family. This result implies that some of the couples in this study used an egalitarian strategy by which both careers and the couple’s relationship are of equal importance (Känsälä et al., 2015).

### Theoretical contributions

Following the COR theory, we confirmed that the decision of employed husbands and wives to live apart affects the stay-at-home spouses’ wellbeing in the work, family, and marital domains, mainly because FIW drains their limited resources. Moreover, the commuters’ family commitment as a form of spousal support extended the resource caravans’ perspective of the COR theory and captured the compensatory effect of resources on the FIW–family satisfaction relationship between couples. This is in line with the COR theory’s proposition that resource gain is even more important when one faces resource losses (Hobfoll, 2012), particularly for stay-at-home spouses in the non-cohabitation family context.

However, partner family commitment was only observed in promoting wellbeing in the family domain. We suggest that other resources from work and marital domains should be specifically employed and tested in future research to improve the work and conjugal wellbeing of stay-at-home spouses. For example, job autonomy was found to increase flexibility in the workplace, and intimacy could enhance trust between couples, thus helping them maintain their marriages.

Furthermore, the family condition (cohabitation/non-cohabitation) was tested as a situational factor that might bring commuter couples into two parallel realities, one as a single parent and the other one as a fake single. Future research might utilize border theory (Clark, 2000) or boundary theory (Ashforth et al., 2000) as a framework for permitting a better flow of resources across work and family role boundaries (Allen et al., 2014) and explaining different roles in CMs, specifically to provide more integration between work and family domains among stay-at-home spouses and the physical segmentation among commuters.

In addition, the significant perception of WIF and marriage satisfaction among our commuters correspond with the findings of Lindemann (2017), who reported that commuters emphasize their marriages’ contribution to personal growth and development. In other words, our commuters might have similarly satisfying marriages with their stay-at-home wives who supported their decision to work away from home, thus prompting more WIF for pursuing career development. Future research should address discrepancies in terms of the calling, career commitment, and role resilience for both roles in CMs.

### Managerial implications

The stay-at-home wives in our study were also employed full time and might stress their career development as much as their husbands. Social supports such as supervisor support or instrumental support from the workplace might increase flexibility in stay-at-home spouses’ family care. A slow remedy from commuters is not useful in emergencies. Moreover, the commuters could adopt diverse online services to help deal with family tasks, use remote communication tools to increase involving family issues, and employ services outside to share the burden of the household.

### Limitations and directions for future studies

Our study has some limitations. First, this study relied on self-report measures, which may suffer from common method variance bias (Podsakoff et al., 2003), although the fact the dyad data came from spouses lends some credence to our results. To minimize this, a longitudinal research design should adopt to separate the explanatory variables over time from the dependent variables. In addition, our findings were limited by our measures. Our role commitment measures focused on role expectations may not mirror actual role involvement (e.g., hours in role). Future studies should assess both role expectations and role involvement. We extended existing studies on CMs to a non-Western society; however, the generalization of our findings may be limited by the convenience sample in Taiwan. It is noted that we had 28.7% of couples living with their parents (in-law). The percentage is a little bit higher than 26.8% of married couples living with parents as discovered in an official report of 2016 (Ministry of the Interior [R. O. C.], 2016).^8^ The sample bias might be concerned in our sample even though we recruited our sample from various regions, industries, and organizations and found that the variable of living with parents was not correlated with the dependent variables and had no effect on predicting the dependent variables. Furthermore, there might be social support from parents living together for CMs; however, the filial obligation is an exceptionally high salience in traditional Chinese society (Qi, 2014), and norms of filial duty have also influenced adult children to provide social support to their aging parents, especially in the condition of the declining health of either parent (Silverstein et al., 2006). Future studies should consider the potential social support in commuter families and recruit larger representative samples to allow for the generalizability of our research findings.

## Conclusion

This study investigated conjugal interactions in CMs to explore the relationships between WFC and the wellbeing of stay-at-home spouses (wives) in non-cohabitation marriages using a dyad data analysis. Our model extends the extant research on CMs by showing that stay-at-home spouses (wives) perceive work/family/marriage dissatisfaction due to FIW and perceive work dissatisfaction due to WIF. We also found that commuters’ family commitment, as a partner effect, buffered the FIW–family satisfaction relationship for stay-at-home spouses. This finding is in line with the COR theory, which captures the compensatory effect of resources.

## Data availability statement

The original contributions presented in this study are included in the article/supplementary material, further inquiries can be directed to the corresponding author.

## Ethics statement

The studies involving human participants were reviewed and approved by the Research Ethics Committee, China Medical University and Hospital, Taichung, Taiwan. The patients/participants provided their written informed consent to participate in this study.

## Author contributions

H-PW and Y-MW together conceptualized the contribution and wrote and reviewed the manuscript. Both authors approved the submission of the manuscript.

## Footnotes

1
http://lavaan.org/APIM_SEM/

2
https://davidakenny.shinyapps.io/APIMoM/

3
https://davidakenny.shinyapps.io/Dingy/

4 The second item of marital role commitment equals 0.46, and the first item of parental role commitment equals 0.48.

5 The second item of FIW equals 0.47.

6 The correlations of the two latent variables were divided by the multiplication term of their Cronbach’s alpha coefficients.

7 The corrected correlation between family commitment and marriage satisfaction was 1.03, and the corrected correlation between family satisfaction and marriage satisfaction was 1.03.

8 26.8% was calculated according to the number of 1,699,445 married couples divided by the total number of 6,327,891 adults living with parents.
